# Diagnostic accuracy of ultrasound-derived fat fraction for the detection and quantification of hepatic steatosis in patients with liver biopsy

**DOI:** 10.1007/s10396-024-01472-6

**Published:** 2024-06-25

**Authors:** Yoshiko Nakamura, Masashi Hirooka, Yohei Koizumi, Ryo Yano, Yusuke Imai, Takao Watanabe, Osamu Yoshida, Yoshio Tokumoto, Masanori Abe, Yoichi Hiasa

**Affiliations:** Department of Gastroenterology and Metabology, Ehime University Graduate School of Medicine, 454 Toon, Ehime, 791-0295 Japan

**Keywords:** Attenuation coefficient, Backscatter, Diagnosis, Liver, Ultrasound

## Abstract

**Purpose:**

This retrospective study was conducted to investigate the diagnostic accuracy of ultrasound-derived fat fraction (UDFF) for grading hepatic steatosis using liver histology as the reference standard.

**Methods:**

Seventy-three patients with liver disease were assessed using UDFF and liver biopsy. Pearson’s test and the Bland–Altman plot were used to assess the correlation between UDFF and histological fat content in liver sections. The UDFF cutoff values for histologically proven steatosis grades were determined using the area under the receiver operating characteristic curve (AUROC).

**Results:**

The median age of the patients was 66 (interquartile range 54–74) years, and 33 (45%) were females. The UDFF values showed a stepwise increase with increasing steatosis grade (*p* < .001) and were strongly correlated with the histological fat content (*r* = .7736, *p* < .001). The Bland–Altman plot revealed a mean bias of 2.384% (95% limit of agreement, − 6.582 to 11.351%) between them. Univariate regression analysis revealed no significant predictors of divergence. The AUROCs for distinguishing steatosis grades of ≥ 1, ≥2, and 3 were 0.956 (95% confidence interval [CI], 0.910–1.00), 0.926 (95% CI, 0.860–0.993), and 0.971 (95% CI, 0.929–1.000), respectively. The UDFF cutoff value of > 6% had a sensitivity and specificity of 94.8% and 82.3%, respectively, for diagnosing steatosis grade ≥ 1. There was no association between UDFF and the fibrosis stage.

**Conclusion:**

UDFF shows strong agreement with the histological fat content and excellent diagnostic accuracy for grading steatosis. UDFF is a promising tool for detecting and quantifying hepatic steatosis in clinical practice.

**Graphical Abstract:**

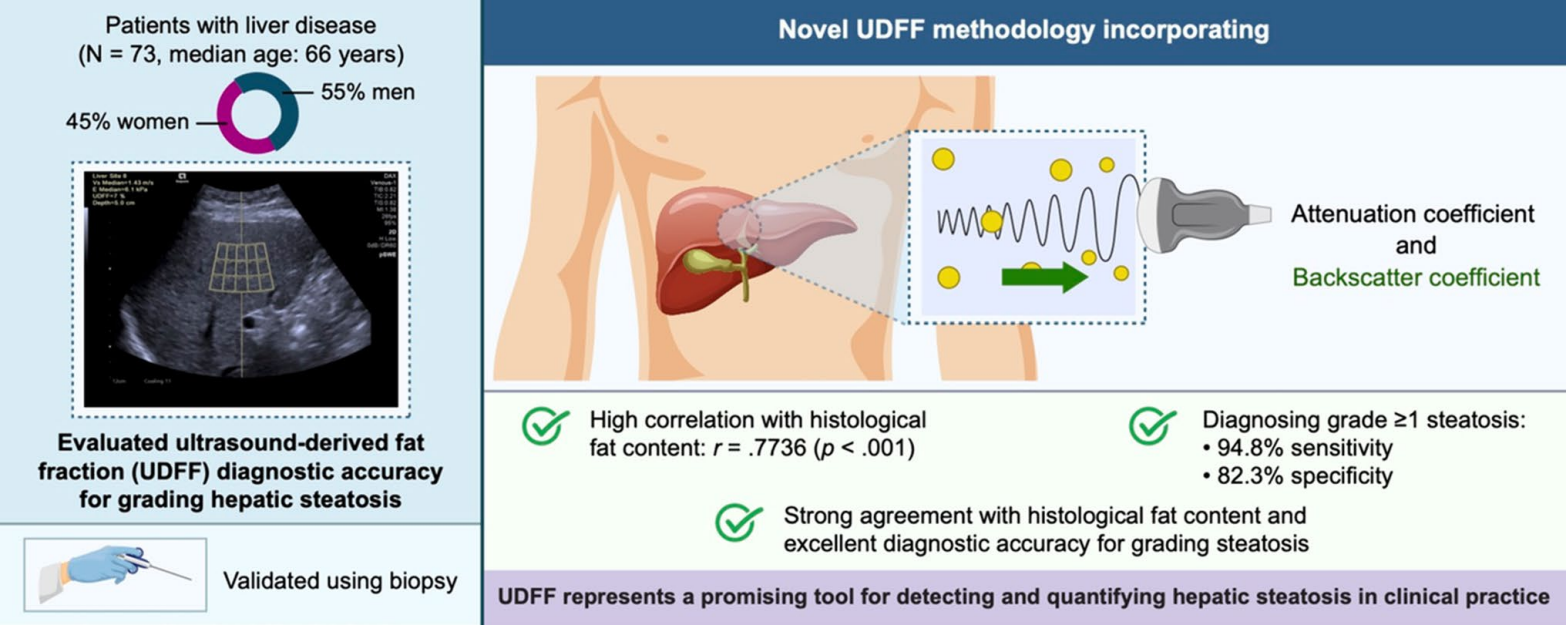

**Supplementary Information:**

The online version contains supplementary material available at 10.1007/s10396-024-01472-6.

## Introduction

Steatotic liver disease, a common manifestation of metabolic syndrome, is a major global health concern. Cirrhosis induced by metabolic dysfunction-associated steatohepatitis is the leading indication for liver transplantation in the United States, and its incidence is expected to increase. Thus, effective screening and monitoring of hepatic steatosis is necessary. Liver biopsy serves as the reference standard for diagnosis and provides valuable information for activity grading and fibrosis staging [1]. However, its use is hampered by the limitations of risk, cost, and resource utilization, according to the American Association for the Study of Liver Diseases guidelines [2].

Imaging has become pivotal for the noninvasive identification and quantification of steatosis. The controlled attenuation parameter (CAP) [3] has been extensively studied and applied clinically. However, challenges in accurate quantification and monitoring of changes have been well-documented [4]. B-mode ultrasound-guided techniques have been introduced for detecting hepatic steatosis to address these shortcomings [5–7].

Fundamental quantitative ultrasound methods, specifically the attenuation coefficient (AC) and backscatter coefficient (BSC), are correlated with the hepatic fat fraction [8–10]. AC has been investigated extensively [11]; however, challenges, such as variability in the thickness of subcutaneous fat and similar cutoff values for moderate and severe steatosis, persist [12, 13].

Ultrasound-derived fat fraction (UDFF), which incorporates AC and BSC, was developed to address these limitations. Dillman et al. [14] reported a strong correlation between UDFF and magnetic resonance imaging (MRI)-proton density fat fraction (PDFF), indicating a high sensitivity for detecting hepatic steatosis using a deep abdominal transducer (DAX; Siemens Healthcare K.K., Tokyo, Japan). DAX transducers can acquire images at diagnostic depths of up to 40 cm, which is advantageous for patients who are overweight or obese. However, reports on its accuracy, using liver biopsy as a reference standard, are scarce. Therefore, this study aimed to evaluate the diagnostic accuracy of UDFF using histology as the reference standard.

## Materials and methods

### Patients

**Fig. 1:**
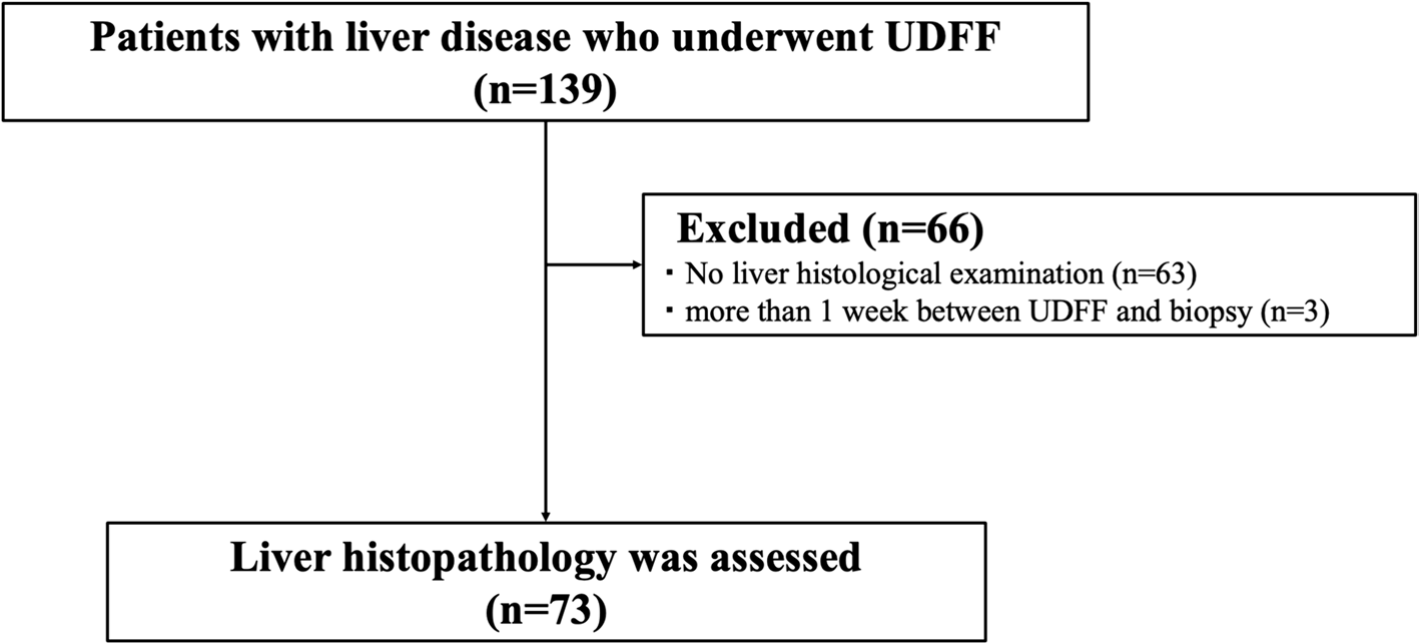
Flowchart showing the patient enrollment process in this study.   *UDFF * ultrasound-derived fat fraction

This study was approved by the Institutional Ethics Committee and conducted in accordance with the principles of the Declaration of Helsinki. Patients with liver disease who were assessed using UDFF between April 2022 and January 2023 were enrolled (Fig. 1). The inclusion criteria were as follows: (1) burden on liver disease and (2) ability to follow breathing control instructions. The exclusion criterion was that a liver biopsy was not performed within 7 days before or after the UDFF examination. The requirement of obtaining informed consent was waived due to the retrospective nature of this study.

### UDFF measurements

UDFF was measured by two operators [M.H. (25 years of experience) and Y.N. (10 years of experience)] using an ACUSON Sequoia ultrasound system (Siemens Healthcare K.K., Tokyo, Japan) equipped with DAX. The DAX transducer is a curved and piezoceramic transducer (1.0−3.5 MHz). Each patient was placed in the supine position, and UDFF was measured after fasting overnight.

**Fig. 2:**
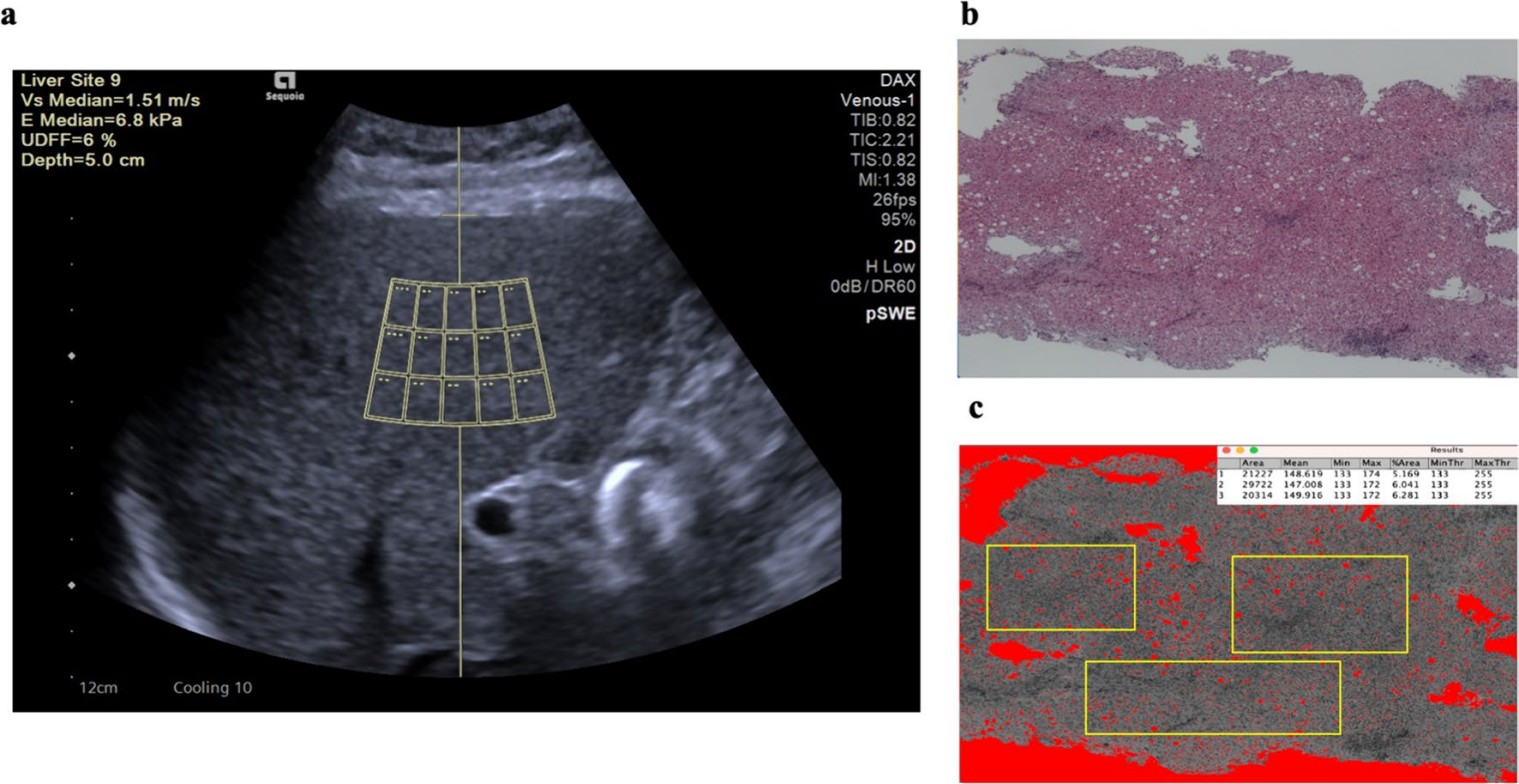
A 64-year-old man with PBC and alcoholic liver disease. **a** UDFF measurement: the median value from the five UDFF measurements was 6%. **b** H&E staining of the liver section shows steatosis grade 1. **c** Quantification of histological liver fat content calculated with image J software: the overall median percentage of five different ROIs was 5.2%.  *PBC * primary biliary cholangitis, *UDFF* ultrasound-derived fat fraction, *ROI* region of interest, *H&E* hematoxylin and eosin

Each patient underwent five separate UDFF measurements. The detection site included segments V–VII and was free of large blood vessels, gallbladder, and liver lesions, as viewed using the intercostal approach. The operator set a crossbar along the liver capsule and then positioned the underlying 3-cm rectangular region of interest (ROI) exactly 1.5 cm deep into the liver capsule (Fig. 2a). Each UDFF measurement was recorded on the captured image and summarized in a manufacturer-generated report on the ultrasound machine. The median values were calculated from five measurements and used here.

### Histological diagnosis

Histological fat content was evaluated using a liver biopsy performed within 7 days before or after the UDFF measurement. The liver biopsy samples were obtained using a cutting needle with a diameter and length of 1.6 mm and 15 mm, respectively. All liver samples were fixed in formalin and embedded in paraffin. The sections (4-µm thick) were stained with hematoxylin and eosin (H&E), impregnated with silver (Fig. 2b), and assessed histologically by two experts (Y.T. and M.A. with 25 and 30 years of experience, respectively). Liver steatosis was scored as follows based on the study by Brunt [15]: grade 0, < 5%; grade 1, 5–33%; grade 2, 33–66%; and grade 3, > 66%. The presence of inflammation, ballooning, and fibrosis was recorded.

H&E-stained liver sections were analyzed to quantify the percentage of fat content using ImageJ software (National Institutes of Health, Bethesda, MD, USA), as described previously [16] (Fig. 2c). Five different parenchymal ROIs were placed on two or three sections per individual; the largest ROIs free of vessels, bile ducts, and tumor lesions were selected. The median values were calculated from five measurements. The fibrosis stage and activity scores were defined using the Meta-analysis of Histological Data in Viral Hepatitis scoring system [17].

### Statistical analysis

Continuous variables are expressed as median (interquartile range [IQR]). The parametric and non-parametric data were compared using the Jonckheere-Terpstra trend and Steel-Dwass tests, respectively. The area under the receiver operating characteristic curve (AUROC) was used to assess the diagnostic performance of UDFF in detecting hepatic steatosis, defined as histological grade ≥ 1. The Youden index was used to define the optimal UDFF thresholds for diagnosing hepatic steatosis.

The sensitivity, specificity, positive predictive value, negative predictive value, positive likelihood ratio (LR+), and negative likelihood ratio (LR-) were calculated to determine the accuracy of UDFF. Linearity and systematic bias were analyzed to assess the agreement between the UDFF values and fat content in the liver tissue. The correlation between the fat content and UDFF measurement was determined using Pearson’s correlation coefficient (r). Bias, defined as the average difference between the fat content and UDFF measurements, was assessed using Bland–Altman analysis. The 95% limits of agreement (LOAs) were calculated and shown in the Bland–Altman plots. The correlation between the difference and mean of the measurements, fixed error, and proportional error were calculated. The points that were not within the bias (± 1.96 standard deviation [SD]) in the Bland−Altman plots were considered divergent. The causes of divergence were analyzed using regression analysis. The correlation of UDFF with the fat content in the liver was assessed using univariate regression analysis. *P-*values of < 0.05 denoted statistical significance. All statistical analyses were performed using STATA version 15.0 (Stata Corp, College Station, TX, USA) or JMP version 11.2.0 (SAS Institute, Cary, NC, USA).

## Results

### Patients

Seventy-three patients (33 women, median age 66.0 years) were included in this study (Fig. 1). Table 1 presents the clinical and serological characteristics of the patients. The median body mass index (BMI) was 24.5 (IQR, 22.2–29.5) kg/m^2^, and the median skin-to-capsular distance (SCD) was 19 (IQR, 16–23) mm. The median HbA1c was 6.1 (IQR, 5.7–7.1)%, and the median of the mean UDFF values was 6.0 (IQR, 4.0–12.5)%.

**Table 1:** Patient characteristics (*n* = 73)

Characteristic	
Age, years	66 (55−74)
Male/female	40/33 (55%/45%)
Etiology	
MASLD/ALD/HBV/HCV/AIH/PBC/others	27/10/8/12/3/6/7
Body mass index, kg/m^2^	24.5 (22.2−29.5)
Skin-capsule distance, mm	19.0 (16.0−23.0)
Platelets, ×10^4^ µL	18.9 (13.5−25.7)
AST, U/L	36 (26−49)
ALT, U/L	34 (21−51)
GGT, U/L	47 (30−104)
HbA1c, %	6.1 (5.7−7.1)
Total cholesterol, mg/dL	171 (158−196)
HDL, mg/dL	46 (40−53)
LDL, mg/dL	92 (80−114)
Triglycerides, mg/dL	92 (72−120)
M2BPGi, COI	1.26 (0.86 − 3.02)
Mean UDFF, %	6 (4 − 12)
Histologic features Steatosis grade;	
S0/1/2/3	34/25/11/3
Fibrosis stage;	
F0/1/2/3/4	11/23/12/11/16

Data are expressed as the median and interquartile range or n (%)

*MASLD* metabolic dysfunction-associated steatotic liver disease, *ALD* alcoholic liver disease, *HBV* hepatitis B virus antigen positive, *HCV* anti-hepatitis C virus positive, *AIH* autoimmune hepatitis, *PBC* primary biliary cholangitis, *AST* aspartate aminotransferase, *ALT* alanine aminotransferase, *GGT* g-glutamyl transferase, *HbA1c* hemoglobin A1c, *HDL* high-density lipoprotein, *LDL* low-density lipoprotein, *M2BPGi* Mac-2 binding protein glycosylation isomer, *UDFF* ultrasound-derived fat fraction

### Reproducibility of UDFF and hepatic fat content

**Fig. 3:**
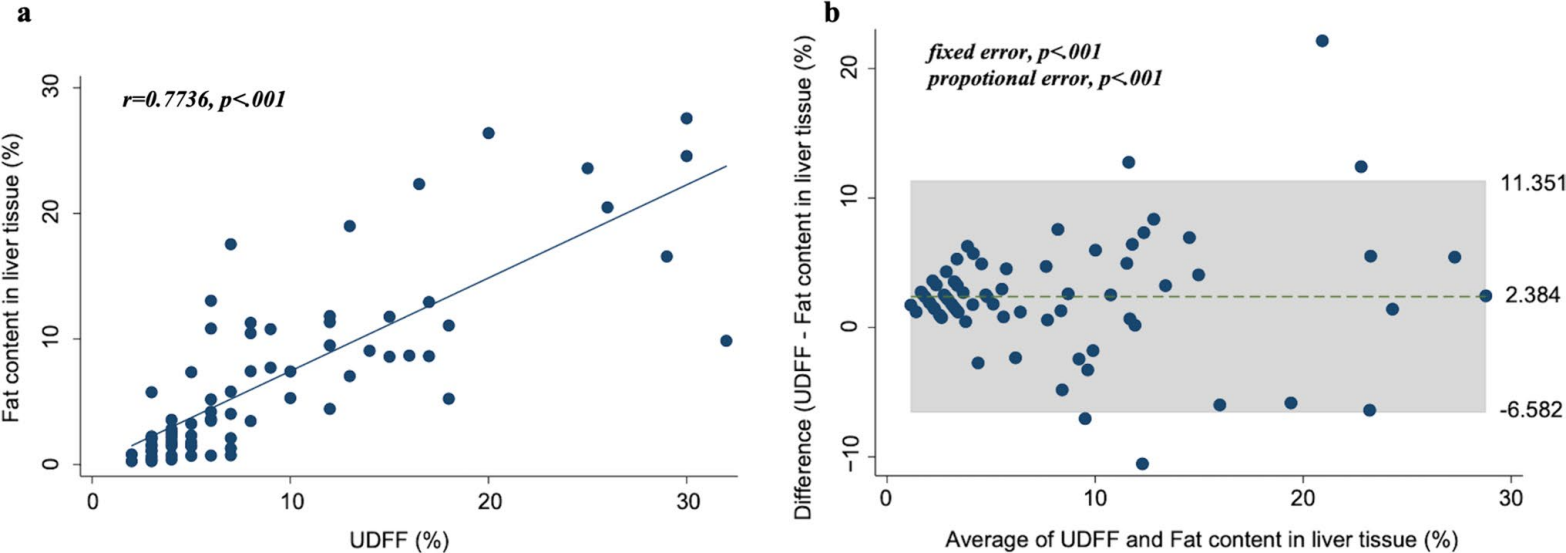
**a** Scatterplot showing the correlation between UDFF and histological fat content in liver tissue. Circles represent patients in this study. **b** Bland–Altman plot showing a comparison of the median UDFF and median histological fat content in liver tissue. The mean bias between them was 2.384%, with a significant fixed error (*p* < .001). Proportional bias was observed with greater overestimation of fat content by UDFF with increasing fat deposition in the liver.  *UDFF* ultrasound-derived fat fraction

The correlations between steatosis grade, percentage of fat content in the liver tissue, and UDFF were analyzed. A significant correlation was observed between the steatosis grade and the percentage of histological fat content using the Jonckheere-Terpstra trend test (**Supp** Fig. 1, *p* < .001). The linearity test showed a significant and strong correlation between the two measurements (*r* = .7736, *p* < .001, Fig. 3a). The Bland–Altman analysis showed a narrow 95% LOA, with a median difference between UDFF and histological liver fat content of 2.384% (Fig. 3b, upper LOA: 11.351, lower LOA: -6.582). The fixed error was *p* < .001. Figure 3b shows the Bland–Altman plots without values between the upper and lower LOA lines that were considered divergent (five cases, 6.8%). Values above the upper LOA were considered overestimations of UDFF or underestimations of fat content in the liver tissue. In contrast, values below the lower LOA were considered underestimations and overestimations of UDFF and fat content in the liver tissue, respectively. The characteristics of five cases with over 95% LOA were as follows: four women, median age: 67.4 years, etiology: metabolic dysfunction-associated steatotic liver disease, *n* = 3, BMI: 27.5 kg/m^2^, SCD: 18 mm, alanine aminotransferase level: 23 U/L, Mac-2 binding protein glycosylation isomer: 0.95 COI, fibrosis stage: F1 (*n* = 4) and F2 (*n* = 1), and steatosis grade: S1 (*n* = 2) and S2 (*n* = 3). Univariate regression analysis was performed to identify the reason for divergency for each value above or below the LOA. The contributions of BMI and SCD to the divergence were assumed; however, no significant factors, including these factors, were identified **(**Table 2).

**Table 2:** Variables predicting divergence between UDFF and fat content in the liver

Variable	Odds ratio (95% CI)	*p*-value
Female	5.379 (0.571–50.701)	0.142
Age	1.025 (0.957–1.098)	0.478
BMI	1.063 (0.918–1.231)	0.412
SCD	1.011 (0.862–1.19)	0.893
MASLD vs. non-MASLD	2.935 (0.458–18.82)	0.256
Fibrosis stage	0.612 (0.280–1.338)	0.219
Activity grade	0.909 (0.299–2.761)	0.866
ALT	0.983 (0.938–1.029)	0.459
Platelets	1.018 (0.965–1.072)	0.518

*CI* confidence interval, *BMI* body mass index, *SCD* skin-to-capsule distance, *MASLD* metabolic dysfunction-associated steatotic liver disease, *ALT* alanine aminotransferase

#### Diagnostic performance of UDFF

**Fig. 4:**
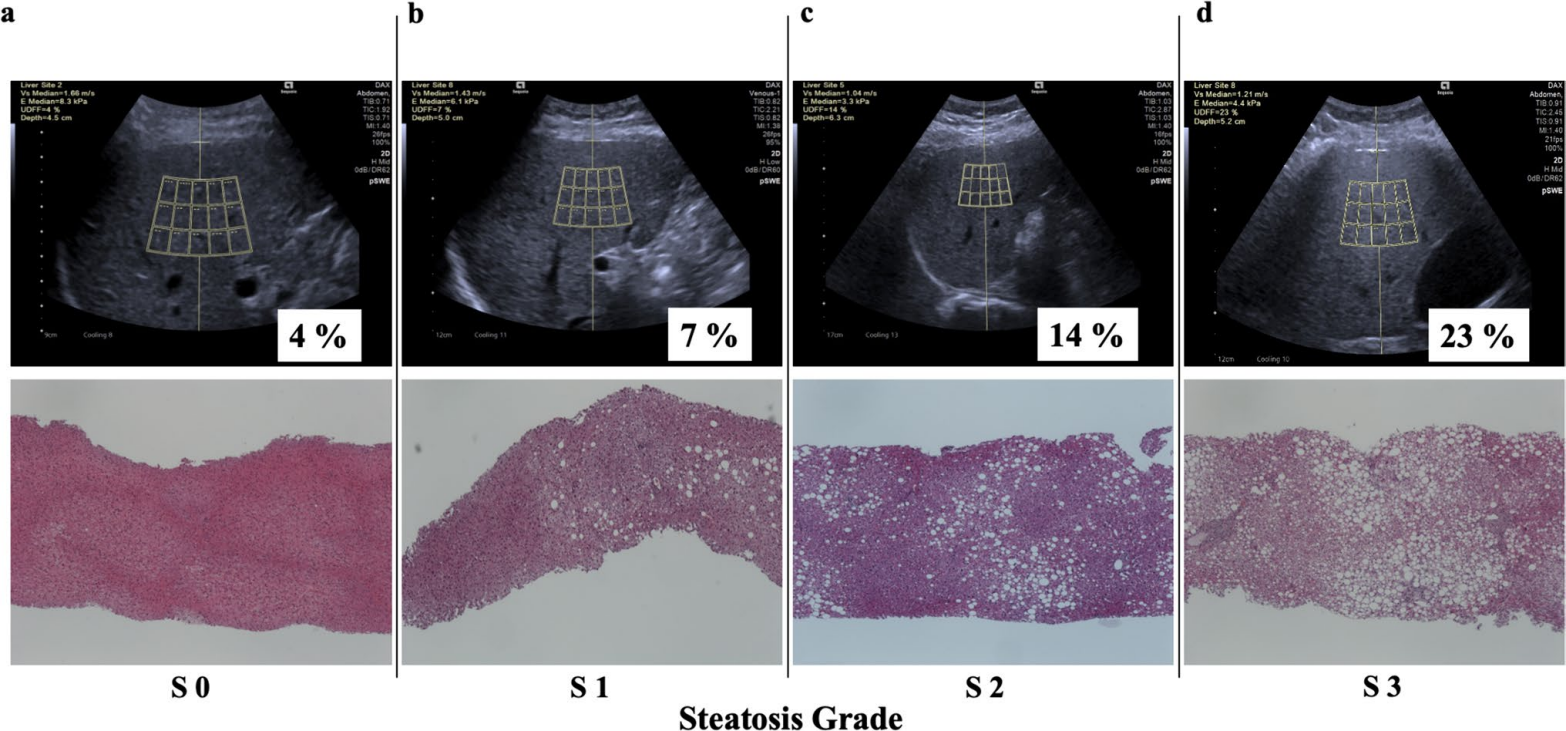
Representative cases. **a** Steatosis grade 0 with 4% of UDFF measurement. **b** Steatosis grade 1 with 7% of UDFF measurement. **c** Steatosis grade 2 with 14% of UDFF measurement. **d** Steatosis grade 3 with a UDFF value of 23%.  *UDFF* ultrasound-derived fat fraction

**Fig. 5:**
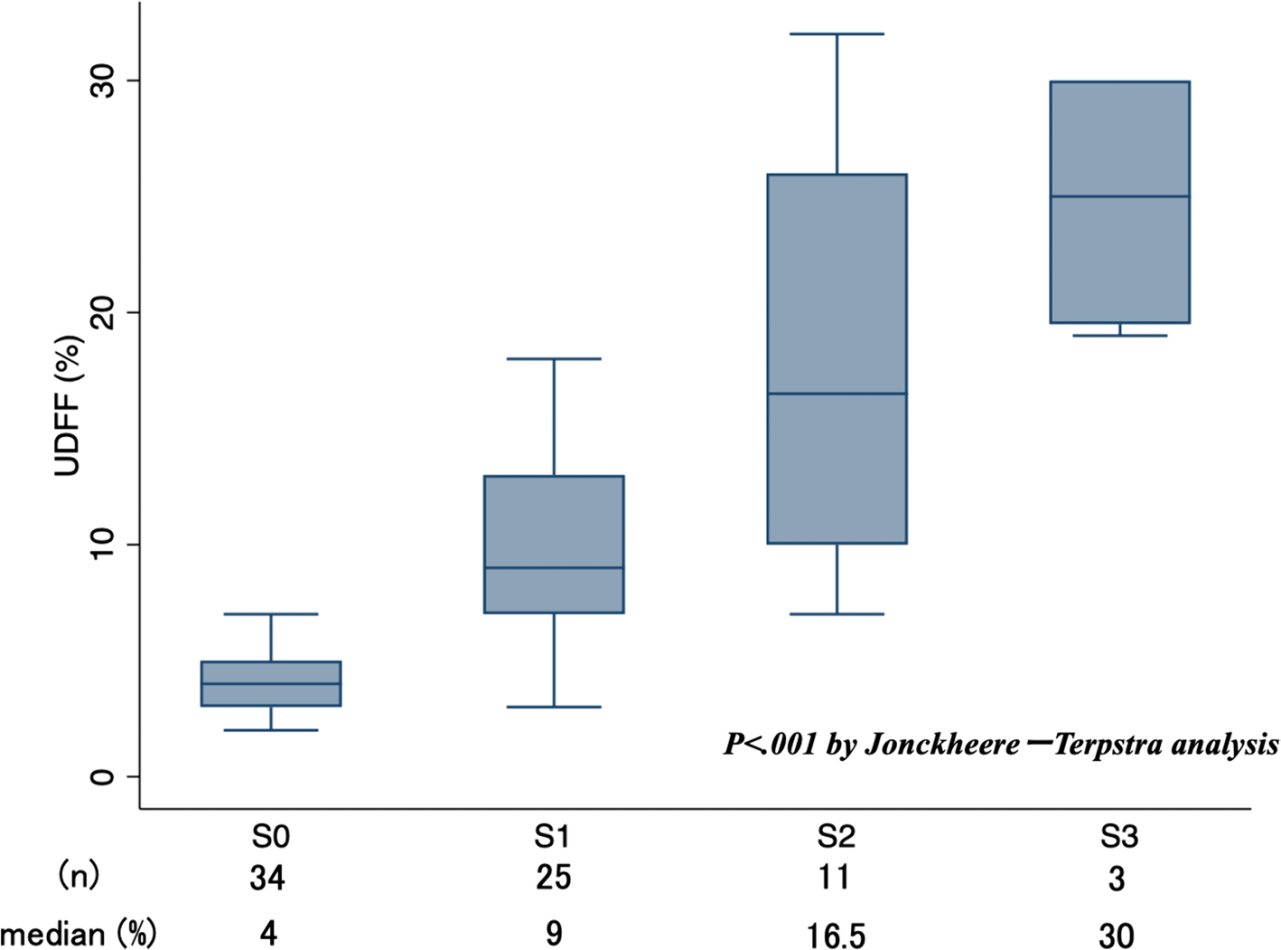
UDFF (%) vs. steatosis grade. Distribution of the UDFF values was classified according to the histological steatosis grade. The Jonckheere−Terpstra trend test yielded a *p-*value of < 0.001.  *UDFF* ultrasound-derived fat fraction

The patients were classified into four groups (grade 0 (*n* = 34), grade 1 (*n* = 25), grade 2 (*n* = 11), and grade 3 (*n* = 3)) according to the histological degree of steatosis. The median UDFF measurements were 4 (IQR, 3–5)%, 9 (IQR, 7–13)%, 16.5 (IQR, 10–26)%, and 30 (IQR, 20–30)% for grades 0, 1, 2, and 3, respectively (Figs. 4 and 5). The median UDFF showed gradual increments with increasing steatosis grade, and a significant correlation was observed between UDFF and the steatosis grade (*p* < .001).

**Fig. 6:**
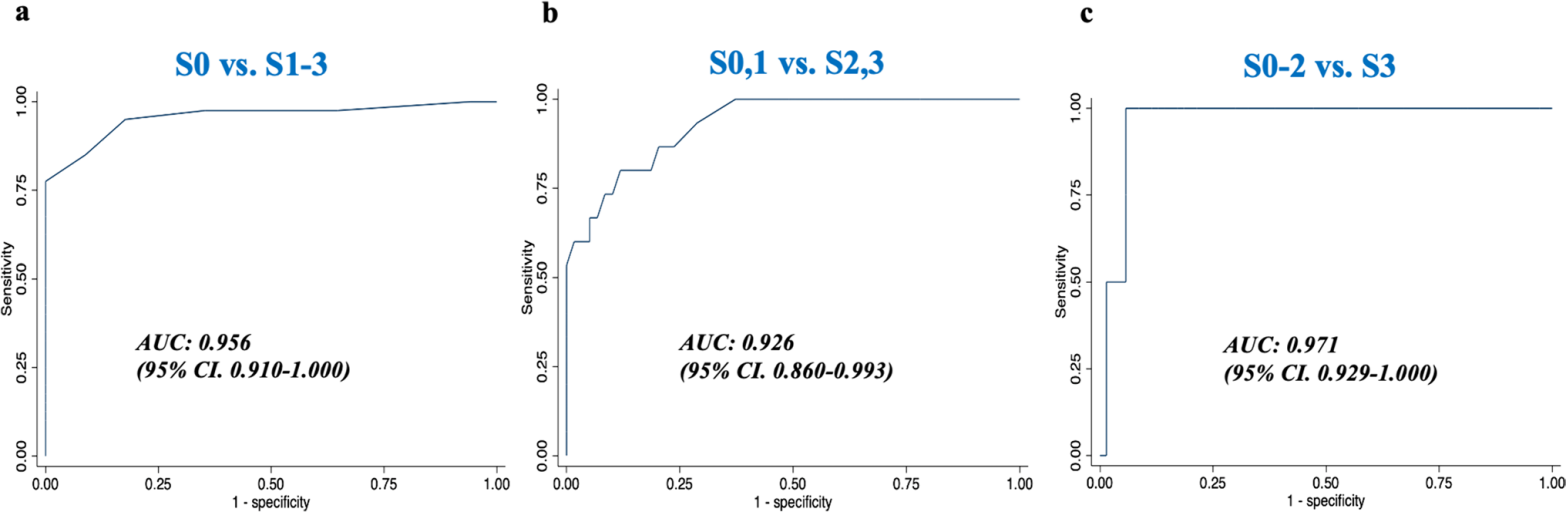
AUROCs of UDFF for diagnosing steatosis. **a** Grades of ≥ 1. **b** Grades of ≥ 2. **c** Grade 3.  *AUROC* area under the curve receiver operating characteristic, *UDFF* ultrasound-derived fat fraction

The AUROCs of UDFF for the diagnosis of grades 1, 2, and 3 were 0.956, 0.926, and 0.971, respectively (Fig. 6). Table 3 shows the diagnostic values of UDFF. According to the Youden index, the cutoff value for a UDFF grade of 1 was 6%, with a sensitivity and specificity of 94.9% and 82.4%, respectively. For UDFF grades of ≥ 1, the cutoff value for a sensitivity of ≥ 90% was 6%, and that for a specificity of ≥ 90% was 7%. The cutoff value for UDFF grades of ≥ 2 was 13%, with a sensitivity and specificity of 78.6% and 88.1%, respectively. The cutoff value for a sensitivity of ≥ 90% was 8%, and that for a specificity of ≥ 90% was 15%. The cutoff value for UDFF grade 3 was 20.0%, with a sensitivity and specificity of 100% and 94.3%, respectively. The cutoff value for a sensitivity of ≥ 90% was 20%, and that for a specificity of ≥ 90% was 18%.

**Table 3:** Diagnostic performance of UDFF for hepatic steatosis

Cutoff	Se	Sp	PPV	NPV	LR+	LR-
S1						
6^*^	94.9	82.4	86.0	93.3	5.38	0.06
6†	94.9	82.4	86.0	93.3	5.38	0.06
7‡	84.6	91.2	91.7	83.8	9.59	0.17
**S2**						
13^*^	78.6	88.1	61.1	94.5	6.62	0.24
8†	92.9	71.2	43.3	97.7	3.22	0.10
15‡	71.4	91.5	66.7	93.1	8.43	0.31
**S3**						
20^*^	100.0	94.3	42.9	100	17.5	0
20†	100.0	94.3	42.9	100	17.5	0
18‡	100.0	91.4	33.3	100	11.7	0

* Cutoff by Youden index, † Cutoff for a sensitivity of ≥ 90%, ‡ Cutoff for a specificity of ≥ 90%

*Se* sensitivity, *Sp* specificity, *PPV* positive predictive value, *NPV* negative predictive value, *LR+* positive likelihood ratio, *LR-* negative likelihood ratio

### Associations between UDFF and other parameters

The interactions between UDFF and other histological findings were assessed. The number of patients with grades 2 and 3 was small; therefore, the interaction between UDFF values and F stage in patients with grade 0 or 1 disease was examined. No significant differences were observed for any of the combinations during the multiple comparisons (**Supp** Fig. 2). The interactions with activity grading were analyzed. No significant interactions were observed for any combinations (**Supp** Fig. 3). Moreover, the linearity test revealed no significant correlation between UDFF and shear wave elastography (m/s) within the same ROI (*r* = − .0918).

## Discussion

This retrospective study demonstrated the high accuracy of UDFF for detecting and grading hepatic steatosis. To the best of our knowledge, no other researchers have reported the high diagnostic performance of UDFF in detecting histologically proven steatosis in patients with liver disease. Furthermore, an excellent correlation was observed between UDFF and liver fat content, which reflects steatosis grade.

CAP, which received FDA approval in 2010, has been widely adopted, and over 160 studies have used it for liver steatosis assessment as of January 2021 [18]. The utility of CAP values in distinguishing between patients with and without steatosis has been established; however, considerable variability exists in the cutoff values for detecting steatosis, particularly across different causes of liver disease [19–22].

Some ultrasound vendors have introduced software to quantify liver fat content based on the AC on B-mode ultrasound imaging. B-mode attenuation liver measurement is expected to provide more accurate values as it can be performed while imaging the measurement site in real time [23, 24]. Several prior studies have shown their excellent performance in improving the accuracy and variability of AC. However, some issues have been raised; for instance, patients with thicker subcutaneous fat and higher BMI show deviations [6, 12]. Moreover, values close to the cutoff points for moderate and severe steatosis indicate that therapeutic intervention is warranted. Ferraioli et al. suggested that it is important to maintain standardized units across all vendors [18]. Although results are typically expressed as decibels per centimeter per megahertz, it is advisable to enhance clinical applicability by creating models that calculate standard deviation and convert the results into a fat percentage, using MRI-PDFF as the reference standard. A previous meta-analysis revealed that the diagnostic accuracy for grade 1 was good for patients with only AC (AUC: 0.89, sensitivity: 0.75, specificity: 0.86). The pooled LR + and LR- were 5.66 and 0.28, respectively [7]. Although this was not a head-to-head study, the diagnostic performance of AC combined with BSC was superior to that of AC alone. Several previous studies using the UDFF have yielded results similar to ours [25, 26]. Kuroda et al. reported that a multivariable quantitative US approach focusing on the acoustic properties of the ultrasound-guided attenuation parameter, integrated BSC, and signal-to-noise ratio may be effective in discriminating at least 5% steatosis in chronic liver disease [27]. These results suggest that the diagnostic accuracy of fatty liver can be improved by adding other ultrasound parameters to the measurement method instead of using AC alone.

UDFF is a novel method that incorporates both AC and BSC. The resultant UDFF was presented as a percentage (%), analogous to MRI-PDFF [26], but not a percentage of liver fat content. UDFF demonstrated a linear correlation with fat content, indicating that as the UDFF index value increased, the corresponding fat content within the liver also increased. Bland–Altman analysis was performed to assess the agreement between UDFF and liver fat content. A mean divergence of 2.384% between UDFF and histologically quantified fat content was observed, with evidence of a proportional error caused by larger UDFF overestimation as hepatic fat content increased. This result is also similar to that of a previous study: a mean bias of 4.0% was observed between UDFF and MRI-PDFF with a proportional bias [14]. The authors of that study considered that capsular reverberation artifacts may have led to this divergence. Barr et al. presented capsular reverberation artifacts that increased without the incorporation of an offset between the liver capsule and the measurement ROI as attenuation measurements increased [28]. In the present study, attenuation and backscatter were used to determine the UDFF values using the same system reported by Dillman et al. [14]. Therefore, the fixed and proportional biases of overestimation by UDFF should be elucidated in further studies.

Additionally, DAX may have contributed to the good diagnostic performance observed in the present study. The DAX transducer, featuring an advanced multi-D beam formation [29], is capable of imaging at diagnostic depths of up to 40 cm, making it tailor-made for patients with a high BMI. Therefore, it can be advantageous and reliable to perform ultrasound in patients with thicker subcutaneous adipose tissue [29, 30]. The present study comprised some morbidly obese individuals. Based on the results of regression analysis for divergence between UDFF and SCD, DAX could overcome the influence of subcutaneous skin and fat. Few studies have addressed the utility of the DAX transducer among the Japanese population. The thickness of the DAX transducer is 28 mm, which is relatively thicker than the convex ones. This may pose challenges for operators when performing ultrasound examinations. However, there were no difficulties for operators to scan with the DAX transducer in the present study. Moreover, the UDFF transducer can also be used to evaluate liver stiffness within the same ROI. A previous study showed that liver stiffness measured with DAX was feasible and reliable for Japanese patient populations [31]. The function of simultaneous measurement might be useful for conducting assessments quickly.

Our study had several limitations. First, this was a single-center study. The evidence of the inter- and intra-observer agreements was insufficient as it was evaluated among only 11 patients. The observed differences were 0.418% and 0.227%, respectively. Linearity tests revealed significant correlations between them (*r* = .9927, 0.9979). Moreover, previous study showed that UDFF was a reliable method with high inter- and intra-observer concordance [14]. Our results suggest excellent inter- and intra-observer agreements; however, the number of patients was quite small. Therefore, further studies are necessary to validate the inter- and intra-observer concordance. Second, not all cases were assessed using MRI-PDFF due to accessibility and limitations. Third, a head-to-head comparison of the UDFF and AC measurements alone was not performed in the present study. This is necessary to clarify the advantages of combining AC and BSC. Fourth, the utility of the DAX probe among the Japanese cohort was not assessed. Further studies are required to validate UDFF performance in clinical application of the DAX transducer. Finally, the diagnostic accuracy of UDFF across various liver disease etiologies was not investigated. Therefore, a multicenter prospective study with a larger sample size is required to investigate the diagnostic accuracy of UDFF across multiple etiologies.

## Conclusion

In the present study, UDFF showed a strong positive correlation with the histological steatosis grade. This suggests that UDFF is a promising clinical tool for the detection and quantification of hepatic steatosis.

## Supplementary Information

Below is the link to the electronic supplementary material.

Supplementary file1 (DOCX 14 KB)

Supplementary file2 (TIFF 6596 KB)

Supplementary file3 (TIFF 6596 KB)

Supplementary file4 (TIFF 6596 KB)
