# Supplementary material for: Diagnostic accuracy of ultrasound-derived fat fraction for the detection and quantification of hepatic steatosis in patients with liver biopsy
Source: J Med Ultrason (2001). 2024 Jun 25;52(1):85–94. doi: 10.1007/s10396-024-01472-6 (PMC12988983; doi:10.1007/s10396-024-01472-6)
Supplement: Supplementary file 1 — Supplementary file1 (DOCX 14 KB) [file 10396_2024_1472_MOESM1_ESM.docx]

**Supporting information**

**Supplementary Fig. 1** Steatosis grade vs. fat content in the liver tissue (%). The distributions of fat content in the liver tissue were classified according to the histological steatosis grade. Jonckheere-Terpstra trend test was *p* < .001.

**Supplementary Fig. 2** UDFF (%) vs. fibrosis stage. The distributions of UDFF were classified according to the histological fibrosis stage among **a** steatosis grade 0; **b** steatosis grade 1. There was no significant difference by Steel-Dwass test.

UDFF, ultrasound-derived fat fraction.

**Supplementary Fig. 3.** UDFF (%) vs. activity grade. The distributions of UDFF were classified according to the histological activity grade among **a** steatosis grade 0; b steatosis grade 1. There was no significant difference by Steel-Dwass test.

UDFF, ultrasound-derived fat fraction.
