# Supplementary figures and images for: Diagnostic accuracy of ultrasound-derived fat fraction for the detection and quantification of hepatic steatosis in patients with liver biopsy
Source: J Med Ultrason (2001). 2024 Jun 25;52(1):85–94. doi: 10.1007/s10396-024-01472-6 (PMC12988983; doi:10.1007/s10396-024-01472-6)

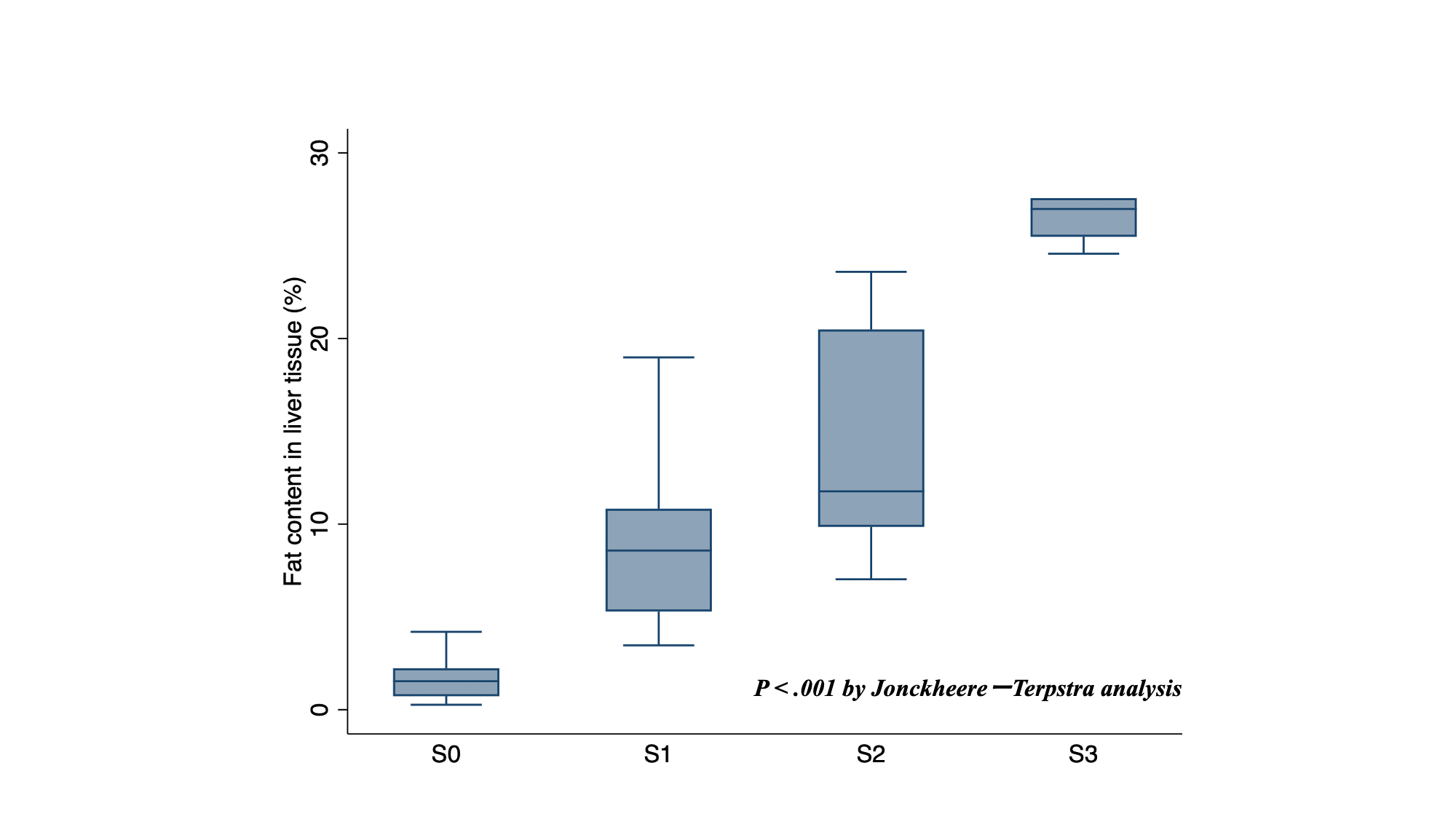

Supplement: Supplementary file 2 — Supplementary file2 (TIFF 6596 KB) [file 10396_2024_1472_MOESM2_ESM.tiff]

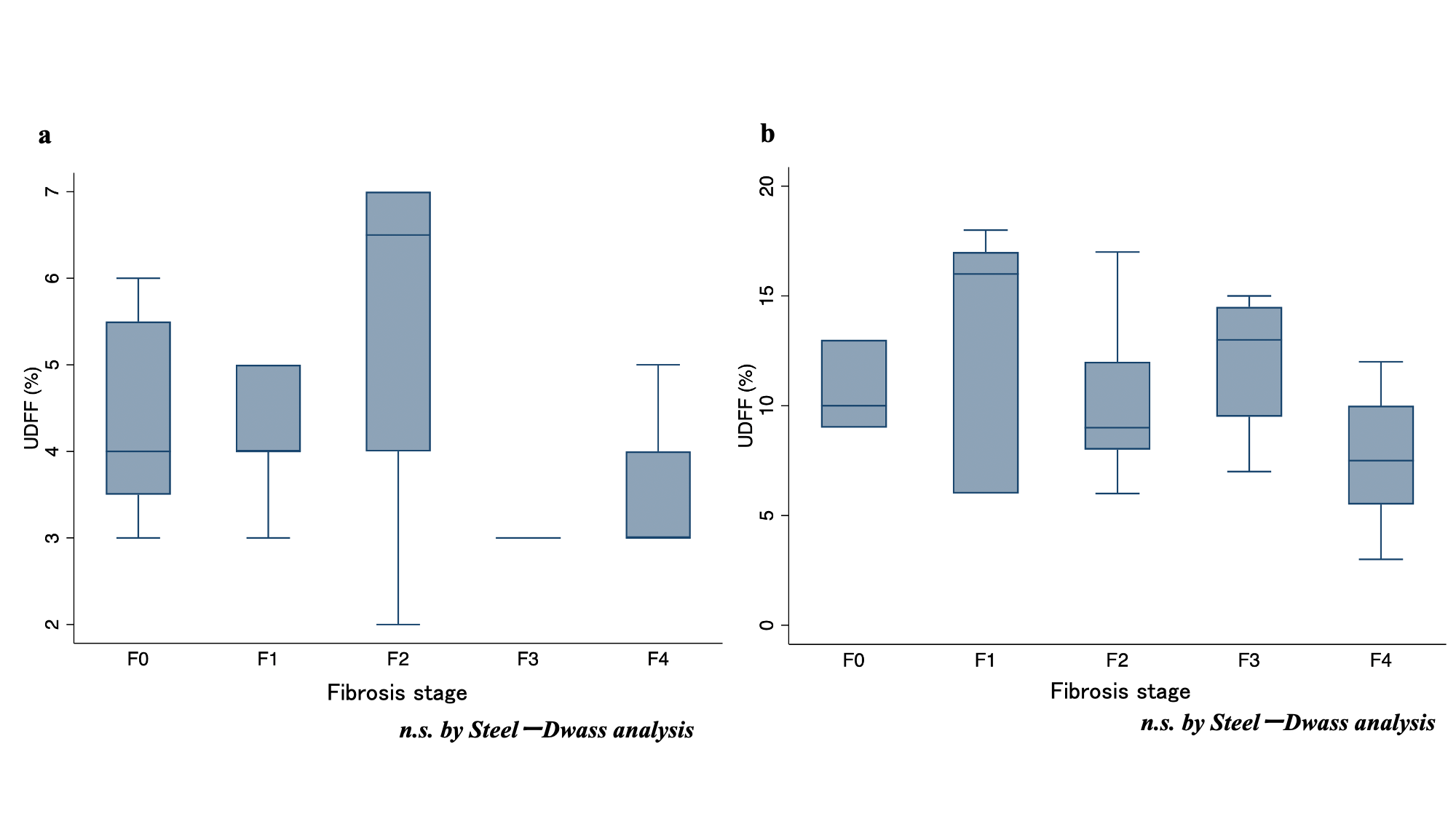

Supplement: Supplementary file 3 — Supplementary file3 (TIFF 6596 KB) [file 10396_2024_1472_MOESM3_ESM.tiff]

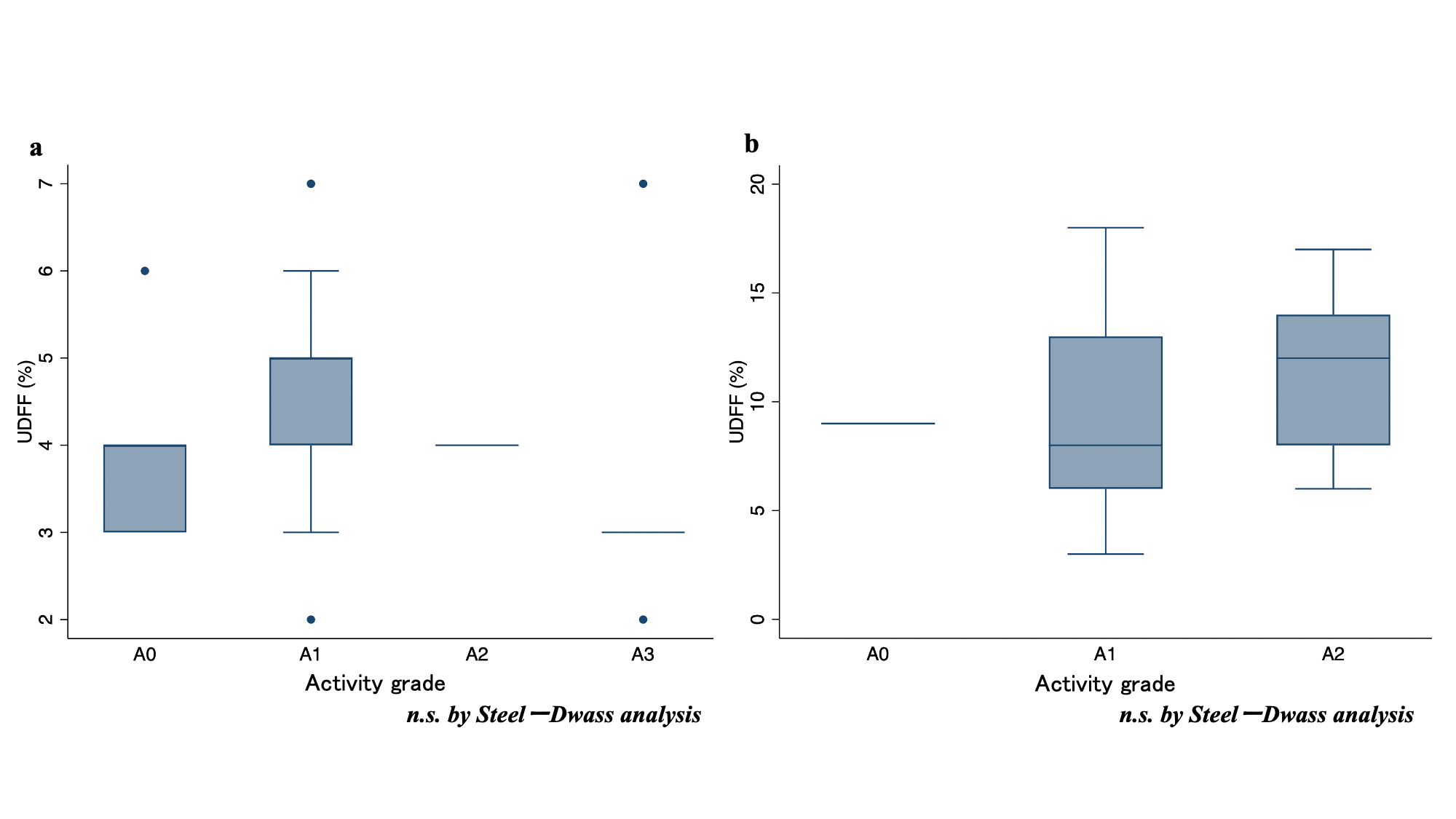

Supplement: Supplementary file 4 — Supplementary file4 (TIFF 6596 KB) [file 10396_2024_1472_MOESM4_ESM.tiff]
